# Determinants for progression from asymptomatic infection to symptomatic visceral leishmaniasis: A cohort study

**DOI:** 10.1371/journal.pntd.0007216

**Published:** 2019-03-27

**Authors:** Jaya Chakravarty, Epco Hasker, Sangeeta Kansal, Om Prakash Singh, Paritosh Malaviya, Abhishek Kumar Singh, Ankita Chourasia, Toolika Singh, Medhavi Sudarshan, Akhil Pratap Singh, Bhawana Singh, Rudra Pratap Singh, Bart Ostyn, Michaela Fakiola, Albert Picado, Joris Menten, Jenefer M. Blackwell, Mary E. Wilson, David Sacks, Marleen Boelaert, Shyam Sundar

**Affiliations:** 1 Department of Medicine, Institute of Medical Sciences, Banaras Hindu University, Varanasi, India; 2 Institute of Tropical Medicine, Antwerp, Belgium; 3 Department of Community Medicine, Institute of Medical Sciences, Banaras Hindu University, Varanasi, India; 4 Cambridge Institute for Medical Research, University of Cambridge, United Kingdom and Telethon Kids Institute, University of Western Australia, Crawley, Australia; 5 Foundation for Innovative New Diagnostics (FIND), Geneva, Switzerland; 6 ISGlobal, Barcelona Ctr. Int.Health Res. (CRESIB), Hospital Clínic-Universitat de Barcelona, Barcelona, Spain; 7 University of Iowa and the Veterans Affairs Medical Centre, Iowa City, Iowa, United States of America; 8 Laboratory of Parasitic Diseases, National Institute of Allergy and Infectious Diseases, National Institute of Health, Bethesda, Maryland, United States of America; Hospital Infantil de Mexico Federico Gomez, UNITED STATES

## Abstract

**Background:**

Asymptomatic *Leishmania donovani* infections outnumber clinical presentations, however the predictors for development of active disease are not well known. We aimed to identify serological, immunological and genetic markers for progression from *L*. *donovani* infection to clinical Visceral Leishmaniasis (VL).

**Methods:**

We enrolled all residents >2 years of age in 27 VL endemic villages in Bihar (India). Blood samples collected on filter paper on two occasions 6–12 months apart, were tested for antibodies against *L*. *donovani* with rK39-ELISA and DAT. Sero converters, (negative for both tests in the first round but positive on either of the two during the second round) and controls (negative on both tests on both occasions) were followed for three years. At the start of follow-up venous blood was collected for the following tests: DAT, rK39- ELISA, Quantiferon assay, SNP/HLA genotyping and *L*.*donovani* specific quantitative PCR.

**Results:**

Among 1,606 subjects enrolled,17 (8/476 seroconverters and 9/1,130 controls) developed VL (OR 3.1; 95% CI 1.1–8.3). High DAT and rK39 ELISA antibody titers as well as positive qPCR were strongly and significantly associated with progression from seroconversion to VL with odds ratios of 19.1, 30.3 and 20.9 respectively. Most VL cases arose early (median 5 months) during follow-up.

**Conclusion:**

We confirmed the strong association between high DAT and/or rK39 titers and progression to disease among asymptomatic subjects and identified qPCR as an additional predictor. Low predictive values do not warrant prophylactic treatment but as most progressed to VL early during follow-up, careful oberservation of these subjects for at least 6 months is indicated.

## Author summary

Visceral Leishmaniasis (VL) or Kala-azar is a vector born disease, deadly if not treated. On the Indian subcontinent VL is caused by the protozoan parasite *Leismania donovani*, transmitted by an insect vector, sand fly of the *Phlebotomus argentipes* species, and considered an anthroponotic disease. Not every *L*.*donovani* infection progresses to clinical VL disease, and only a small minority of those infected will progress to disease and therefore not all those infected need to be treated. Importantly, diagnostic and treatment options have considerably improved over the past 10 years. There are several markers of infection in VL: antibody-tests as DAT, rK39-ELISA, markers of cellular immunity as the Quantiferon assay, and molecular markers as quantitative PCR. Also SNP/HLA genotyping has been shown to be associated with VL. However the factors that determine who will and who will not progress from infection to disease remain largely unknown. To try and elucidate the factors associated with progression to disease we identified a cohort of healthy recently infected persons in a highly VL endemic area of Bihar, India, and followed them up for three years. We also included in the follow-up an age and village matched group of initially seronegative controls. SNP/HLA genotyping was performed on all subjects to identify genetic predisposition. The only factors strongly and significantly associated with progression to disease turned out to be high DAT and/or rK39 titers and positive qPCR. The proportion progressing to disease was too low to merit preventive treatment. As most disease tends to occur early during follow-up, it is recommendable to closely follow up those with high antibody titers or testing qPCR positive over at least a 6-months period.

## Introduction

Visceral leishmaniasis (VL) or kala-azar is the severest form of leishmaniasis and fatal if left untreated. More than 90% of global VL cases occur in just six countries: India, Bangladesh, Sudan, South Sudan, Brazil and Ethiopia [1]. India accounts for approximately 50% of the global burden of VL and is a signatory to a Tripartite Memorandum of Understanding (MoU) to achieve VL elimination from the South-East Asia Region (SEAR). The goal is to reduce the annual incidence of VL to less than 1 case per 10,000 population at the sub-district (*block*) level [2, 3]. This elimination target is expressed as a number of new clinical cases of VL per person-year. However, it is established that many *L*. *donovani* infections do not lead to a clinical episode of VL and that asymptomatic infections far outnumber the clinical cases [4]. A prospective study in India and Nepal showed a ratio of incident asymptomatic infection, measured by recent conversion in antibody tests, to clinical disease of 9 to 1 while in neighboring Bangladesh it was 4 to 1 [5, 6]. Mathematical modeling has suggested that transmission of *L*.*donovani* could be maintained by asymptomatically infected hosts [7, 8]. Therefore the study of asymptomatic infection is considered a key research priority to support the VL elimination initiative [9]. Currently, xenodiagnosis studies are ongoing in India and Bangladesh to establish whether asymptomatic carriers of *L*.*donovani* infection are infectious to sand flies, but there are other issues to address as well.

Mathematical modeling has suggested that detecting and treating clinical cases early enough is key to reducing their transmission potential [10, 11] and therefore, it would be useful if one could identify the infected persons who are most likely to progress to clinical disease. To date it is not established which are the best predictors for the development of active VL disease in somebody with a positive leishmanial infection marker but no signs and symptoms. A strong association has been observed between high baseline antibody titers and progression to VL in the subsequent 36 months in large cohort studies in India, Nepal and Bangladesh [12, 13]. In the above mentioned studies in India and Nepal, even stronger associations for progression to VL were observed with recent seroconversion to high antibody titers.

Whether an infection remains asymptomatic or progresses towards VL probably results from the complex interaction between genetic susceptibility and immune response of the host, combined with parasite, socioeconomic and demographic factors. A genome-wide association study (GWAS) carried out in India showed that HLA class II alleles, in particular, HLA-DRB1, are major genetic risk factors for VL. Sequence-based classical HLA typing and haplotype analysis suggest that risk allele(s) in India belong to HLA-DRB1*13/*14 allele groups and protective alleles to HLA-DRB1*15 allele group [14]. However, the relative contribution of these different factors to the development of VL is still not well understood. We assessed immunological and genetic markers for progression to active clinical disease in a large prospective cohort study in Bihar, India. Our aim was to identify markers that are predictors of progression from *L*.*donovani* infection to clinically symptomatic VL.

## Methods

We conducted this prospective study in two high VL incidence areas of Muzaffarpur district, Bihar State, India from 2008–2015.

### Ethical considerations

The review committee of the U.S. National Institutes of Health (NIH), as well as the Institutional Review Boards of the Institute of Medical Sciences, Banaras Hindu University, Varanasi, India, Institute of Tropical Medicine, Belgium and the University of Iowa reviewed the study protocol and gave ethical clearance for this study. Data was anonymized. All subjects provided written informed consent; in case of illiterate subjects, a thumb print plus a signature of an independent witness was obtained. For minors under the age of 18 years, informed consent was obtained from a parent or guardian.

### Study population

The study was conducted in two areas. The first area (Area-1) had a total population of 19,634 divided over 11 villages with high VL incidence rates before 2009. Two house to house surveys were conducted in Area-1at a one-year interval between December 2009 and February 2011. All residents above two years of age who were present and gave their informed consent (or whose parents gave consent, for minors) were enrolled in the study. A capillary blood sample was obtained on pre-printed Whatmann filter paper in consenting participants and rK39-ELISA, and DAT tests were performed to detect antibodies against VL. Those individuals testing negative for both the tests in the first sero-surveywere re-tested in the second serosurvey in the following year. We defined seroconverters as subjects negative on rK39-ELISA and DAT in the first sero-survey but positive on either of the two assays during the second survey. For each seroconverter a control who was rK39-ELISA and DAT-negative on both survey rounds was recruited into the study, controls were group matched according to age (<10, 10–18 or >18 years) and village of residence.

An interim analysis in 2010 showed that, due to a declining incidence trend of VL, the target sample size of 600 seroconverters could not be achieved [12]. Therefore we selected an additional study area (Area 2) of 10,729 population living in 1,836 households in 16 geographically scattered villages. Villages were selected based on reported recent high VL incidence levels. In this area two similar serosurveys were conducted six months apart to recruit more seroconverters. This time we recruited four controls for each seroconverter, based on the same matching criteria [12, 15].

### Study procedures

All seroconverters and their controls were interviewed to gather baseline demographic and medical history data, and were clinically examined. At the time of recruitment 5 ml of blood was obtained from seroconverters and controls for the following tests: DAT, rK39-ELISA, Quantiferon assay (IFN-γ release assay), SNP/HLA genotyping and quantitative PCR (qPCR). Both the seroconverters and controls were followed up monthly for the development of clinical symptoms of VL. In case of clinical suspicion (i.e. more than 2 week fever history and rK39 RDT positivity), VL was confirmed parasitologically by splenic smear and treated with Amphotericin B as per national guideline recommendation [16]. All the participants were followed up for a minimum of 3 years.

### Laboratory tests

#### Direct agglutination test (DAT)

The standard procedure was followed as described elsewhere [17]. Briefly 100 μl of 1:400 diluted serum samples were serially diluted up to 1:51,200 in V-shaped, microtitre plates in DAT diluents with one positive and one negative control run every fifth plate. Wells in the last row were kept for antigen control. 50 μl of DAT antigen was dispensed to every well [18]. Plates were covered, shaken gently and incubated overnight at room temperature. The DAT results were read against a white background and samples with a titer ≥1:1600 were considered positive.

#### rK39-ELISA

rK39-ELISA was performed as described elsewhere [19]. The optical density (OD) measurements were undertaken at 450nm using a microtitre plate ELISA reader (Molecular Devices, USA). A positive (parasitologically confirmed VL case) and a negative control (filter paper eluate from non-endemic healthy control, NEHC) were run in each plate and the positive control was used as a reference to calculate a relative value of positivity of each sample. Results were expressed as the subject’s optical density (OD) value divided by the OD value of a positive control serum sample ×100, and called percentage point positivity (pp) of a positive control. PP was log transformed to compensate for skewed distribution. The resulting standard value 14 percent of the OD of a positive control was considered for deciding the positivity of samples (cut-off decided for Indian population) [12].

#### IFN-γ release assay (IGRA)

A whole blood assay for detection of antigen-specific IFN-γ production in vitro, and its use in identifying asymptomatically infected individuals in Bihar, has been described [20, 21]. Briefly, 3 ml heparinized whole blood was dispensed in tubes as 1 ml each and incubated PBS, SLA and positive control (phytohemagglutinin, PHA). After 20–24 hr incubation at 37°C, the supernatant (approx 400 μl) were collected from each well and stored for measurement of IFN-γ concentration by ELISA. Antigen-specific IFN-γ levels (expressed as IU/ml) produced in response to SLA stimulation were determined by subtracting background levels measured in the non-stimulated (NIL, PBS) samples. The result was considered positive when the IFN-γ concentration in the antigen wells is >0.78 IU/mL; this cutoff was determined based on the optimal sensitivity (85%) and specificity (100%) by a receiver operating characteristic (ROC) curve constructed from previous data [19].

#### Quantitative PCR (qPCR)

Parasite quantification by real-time polymerase chain reaction (qPCR) was carried out from buffy coat (isolated from blood collected in citrate tube) [22]. Briefly, DNA was extracted using Qiagen DNA extraction kit and TaqMan based qPCR was performed using specific kDNA primes on each DNA sample in duplicateon an Applied Biosystem (ABI)7500 platform (22). For absolute quantification of parasite numbers in the samples, the standard curve method was performed as described previously [22, 23].

#### SNP genotyping

SNP genotyping using a TaqMan based assay was performed as a surrogate method for the HLA-DRB1 screening of our study population at 2-digit level specificity. In our previous genome-wide association [14] and follow-up imputation studies, we have shown that the rs9271255-G allele perfectly correlates with the HLA-DRB1*01/*15/*16 allele groups which are associated with protection against VL. Here, we genotyped the HLA-DRB1-tagging SNP rs9271252 which is in perfect linkage disequilibrium with rs9271255 (r^2^ = 1 in Indian population), and can thus be used as a surrogate marker for determining the key risk versus protective HLA-DRB1 alleles. All the samples were genotyped on ABI 7500 real-time PCR platform using 20ng of purified gDNA per well. For SNP genotyping three levels were considered based on presence or absence of a protective HLA-DRB1 allele. Subjects could either be homozygous for non-protective alleles, heterozygous or homozygous for the protective alleles.

### Quality control

As a quality control measure for each seroconverter identified both the original sample and the sample of the follow-up survey were rerun on the same plate for rK39 ELISA as well as for DAT. Subjects who were intially classified as seroconverters, but for whom quality control serologic testing disagreed with the initial baseline negative or follow-up survey positive results, were kept in the cohort but were reclassified as controls. The assessment of the association between seroconversion and disease was done comparing the final validated set of seroconverters to all controls, as well as by comparing the final set of seroconverters to only the original controls.

### Data analysis

To determine the probability of progression to disease as a function of baseline status for various markers, we calculated odds ratios and confidence intervals using logistic regression. The factors on which converters and controls had been matched, i.e. village of residence and age group, were included in the models. Persons who developed VL before their inclusion in the cohort were excluded from the main analysis. For baseline DAT and rK39 results, we constructed Kaplan-Meier survival plots after subdividing both markers into three categories. DAT titers were regrouped on a 0 to 8 scale, each step representing an increase of one titer step from undiluted (0), via 1:400 (1) up to 1:25,600 or above (8). As cut off for being labeled DAT positive we chose a titer of 1:1600 or above based on our prior studies of subjects in this area [12]. For further analysis we defined three categories, DAT negatives (titer < 1:1,600), moderately DAT positives (titer ≥1:1,600 but < 1:25,600) and strongly DAT positives (titer ≥ 1:25,600). For rK39 ELISA we used percentage points optical density. As we previously defined for subjects in the region, above 14 percentage points was considered positive. For further analysis we divided subjects into three categories based on percentage points (pp) optical density. Titers of ≤ 14 pp were considered negative, >14 pp up to ≤40pp was considered moderately positive, above 40 pp as strongly positive.

## Results

Altogether 1,606 subjects were enrolled, including 476 seroconverters and 1,130 controls. Among the 1,130 controls, 79 were originally classified as seroconverters but re-classified after quality control. Altogether 978 subjects (61%) were female; the proportion was the same among converters and controls. The youngest subjects were two years of age; the oldest was 88 years. The median age of the group of seroconverters was 25 years as compared to 24 years for controls.

Over an average 52 months of follow-up, 17 persons developed VL, eight in the group of seroconverters and nine among controls, resulting in an odds ratio of 3.1 (95% CI 1.1–8.3). Most cases arose early during follow-up, with a median follow-up duration of 5 months and a maximum of 50 months. Fifteen out of 17 cases occurred in area 2, the area with the highest reported incidence at the time of the baseline survey. The association between seroconversion and progression to disease was only observed in this region (OR 3.5, 95% CI 1.2–9.8), whereas in Area 1 there was no association, the odds ratio was 1.3 (95% CI 0.08–21.1).

**Fig 1:**
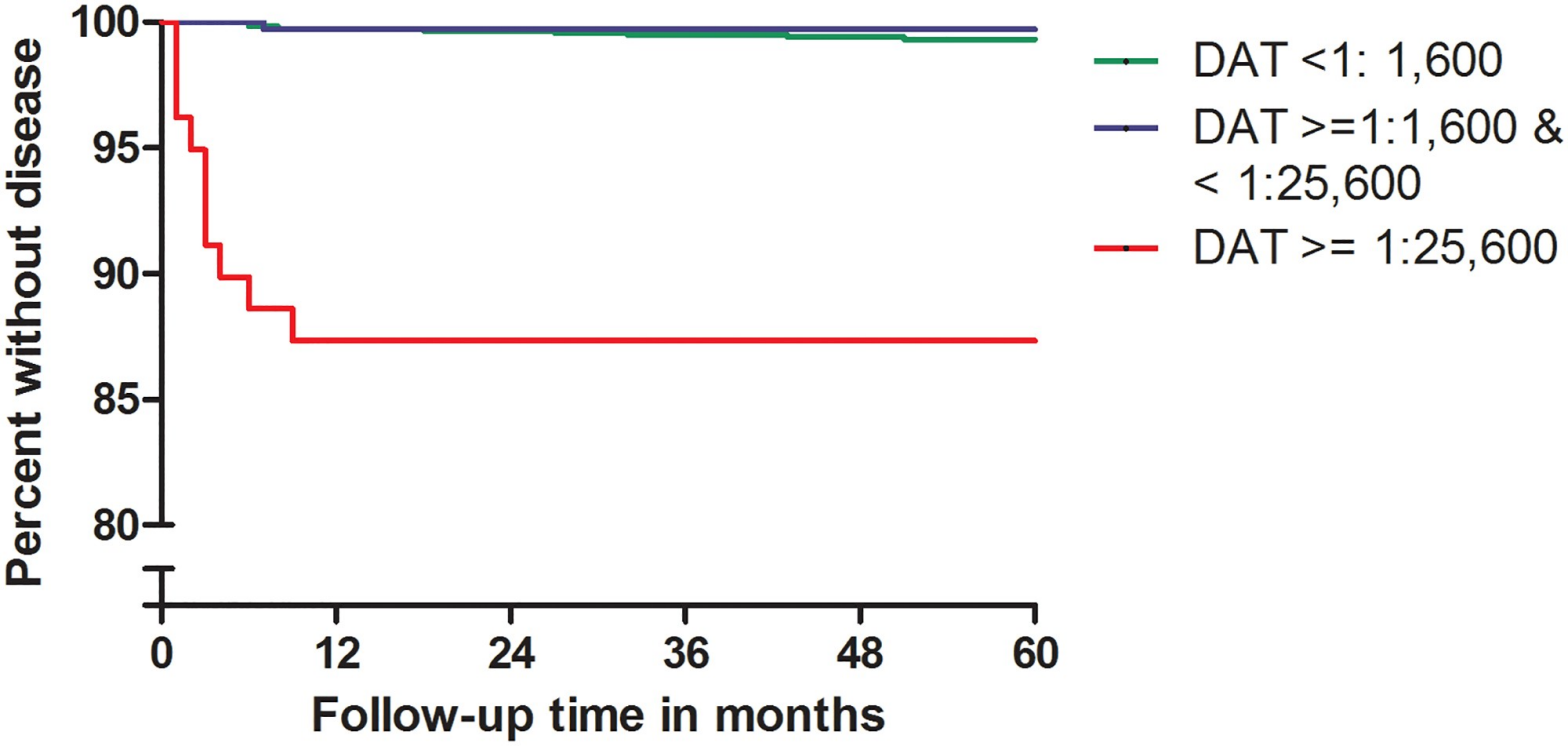
Risk of progressing to disease as a function of initial DAT titer.

Fig 1 shows the probability of progressing to VL independent from the case/control group status. When analyzing all subjects together–seroconverters as well as non-seroconverters-there was a strong association between DAT at the start of follow-up and progression to disease. In the persons with high DAT antibody titers, 8 out of 77 subjects (10.4%) developed VL, all within nine months of follow-up, resulting in an odds ratio of 19.1 (95% CI 4.4–57.1) when compared to DAT negatives. In the latter category only 8 out of 1,175 (0.7%) developed VL.

**Fig 2:**
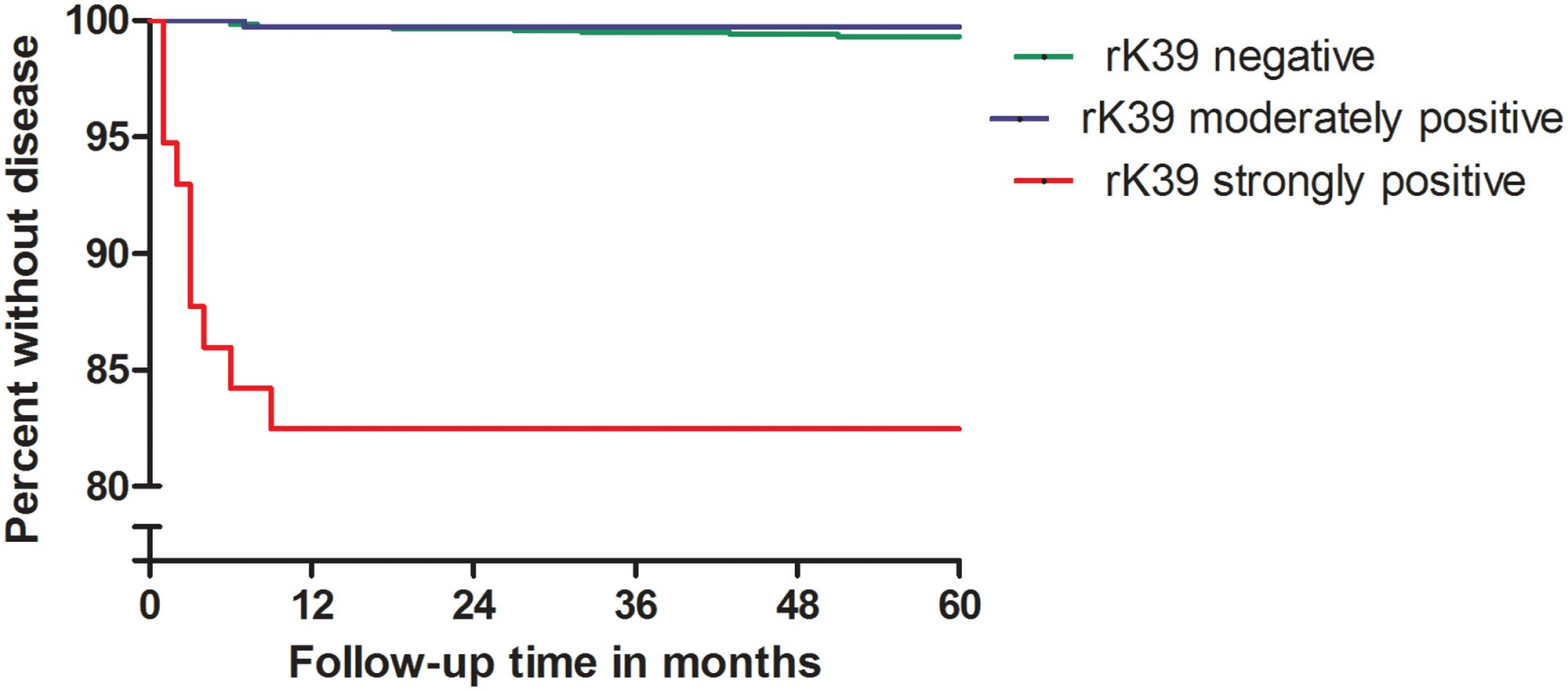
Risk of progressing to disease as a function of initial rK39 ELISA level.

For rK39 ELISA, findings were similar as for DAT. Out of 44 subjects belonging to the high titer category, seven (15.9%) developed VL, all within nine months, resulting in an odds ratio of 30.3 (95% CI 9.6–95.2) when compared to rK39 ELISA negatives (Fig 2). In the latter category only 9 out of 1,416 (0.6%) developed VL.

Among 1,579 subjects tested with Quantiferon (IGRA assay) at the start of follow-up, 280 (17.7%) tested positive. Five out of sixteen VL cases occurred in this group, resulting in an odds ratio of 1.8 (95% CI 0.6–5.3).

Out of 1,604 subjects tested with qPCR, 68 were positive, i.e. exceeded the—1 parasite genomes/ml of blood. Out of those, six(8.8%) developed VL, compared to 11 out of 1,536 (0.7%) among qPCR negatives, resulting in an odds ratio of 20.9 (95% CI 6.5–66.8). All six progressed to disease within four months, four out of six even progressed within two weeks.

Of 957 subjects subjected to SNP genotyping, 380 (39.7%) were homozygous without the protective allele, 442 (46.2%) were heterozygous, and 135 (14.1%) were homozygous for the protective allele. With the first category (homozygous without the protective allele) as reference category, we found odds ratios of 0.61 (95% CI 0.2–1.8) and 0.62 (OR 0.13–3.1) respectively for the second and third category.

When looking at combinations of the three markers that were strongly associated with progression to disease, i.e. high DAT titers, high rK39 titers and qPCR positivity, we observed that relatively little gain in sensitivity was achieved by combining tests. Results are shown in Table 1. A high titer DAT identified 8 out of the 9 cases that were identified by combining the three markers.

**Table 1:** Numbers of subjects that tested positive at baseline to at least one test- at high cut-off, in several combinations of tests (n = 1,600) and numbers of VL cases that developed among those.

Category	Total positive in at least one of the tests(n = 1,600)	Incident VL cases among test positives (%)
qPCR positive and/or high DAT and/or high ELISA	142 (8.9%)	9 (6.3%)
qPCR positive and/or high DAT	133 (8.3%)	9 (6.8%)
qPCRpositive and/or high ELISA	103 (6.4%)	8 (7.8%)
High DAT and/or highELISA	86 (5.4%)	8 (9.3%)
qPCR positive	68 (4.2%)	6 (8.8%)
High DAT	77 (4.8%)	8 (10.4%)
High ELISA	44 (2.8%)	7 (15.9%)

The strong overlap between baseline high DAT titers, high ELISA titers and qPCR positivity among incident VL cases is also apparent from Table 2 below. This table also shows that for each of these markers VL cases among positives arose early during follow-up, an observation that was already visible in the Kaplan-Meier graphs. All cases among subjects that were not highly DAT and/or ELISA positive arose only after a minimum delay of six months. Among qPCR negatives the picture was similar though there was one exception of a case arising after just two months of follow-up.

**Table 2:** Delay between baseline screening and time of onset of disease for 17 VL casesin relation to their initial DAT, rK39 and qPCR status.

ELISA titer	DAT titer	qPCR	Delay between baseline and appearance of VL (months)
HIGH	HIGH	POSITIVE	1
HIGH	HIGH	NEGATIVE	2
HIGH	HIGH	POSITIVE	3
HIGH	HIGH	POSITIVE	3
HIGH	HIGH	POSITIVE	3
HIGH	HIGH	POSITIVE	4
NEGATIVE	NEGATIVE	NEGATIVE	6
NEGATIVE	NEGATIVE	NEGATIVE	6
MODERATE	HIGH	NEGATIVE	6
NEGATIVE	MODERATE	POSITIVE	7
NEGATIVE	NEGATIVE	NEGATIVE	8
HIGH	HIGH	NEGATIVE	9
NEGATIVE	NEGATIVE	NEGATIVE	18
NEGATIVE	NEGATIVE	NEGATIVE	27
NEGATIVE	NEGATIVE	NEGATIVE	32
NEGATIVE	NEGATIVE	NEGATIVE	43
NEGATIVE	NEGATIVE	NEGATIVE	51

## Discussion

Our data show very strong associations between being qPCR positive at baseline and subsequent progression to VL (OR 20.8, 95% CI 6.5–66.8), the same applies to having a high DAT titer (OR 19.1, 95% CI 4.4–57.1) or a high rK39-ELISA titer (OR 30.3, 95% CI 9.6–85.2). There was only a moderately strong association between seroconversion and progression to disease, (OR 3.1, 95% CI 1.1–8.3). There was no significant association between progression to disease and positivity in the IGRA test (OR1.8, 95% CI 0.6–5.3). SNP/HLA genotyping showed a trend towards a protective effect of the genes tested, but the association was weak and non-significant. Both heterozygous and homozygous individuals for the protective variants had lower odds of disease when compared to individuals without the protective variants with odds ratios of 0.61 (95% CI 0.2–1.8) and 0.62 (OR 0.13–3.1) respectively.

This study corroborates our previous findings of a strong association between high DAT and rK39 titers and subsequent progression to disease, as well as the findings by Chapman and others in Bangladesh [13, 15].

The main strength of our study is that we followed up a cohort of seroconverters on DAT and rK39-ELISA over a relatively long period, three years, and included a number of other potential markers of infection. We also performed SNP/HLA genotyping. One of the major difficulties in studies of “asymptomatically infected” is that there is no clear consensus about the case definition. *L*.*donovani* infection status can be measured by antibody, antigen or nucleic acid detection or else by markers of cellular immunity in combination with a clinical assessment of signs and symptoms. Asymptomatically infected persons have been defined in various studies as those who show no clinical signs or symptoms of VL but are positive in at least one marker of infection such as the Leishmanin Skin Test (LST), a marker of cell-mediated immunity [24–26]; an antibody detection test as the DAT, rK39 ELISA, or IFAT [5, 6, 12, 27], or a molecular marker as qualitative or quantitative PCR to detect *Leishmania* spp. DNA [5, 28–30]. Given the lack of agreement between these infection markers when measured cross-sectionally, the case definition of “asymptomatically infected” is a recurrent matter of discussion [31].

Medley et al. [11] pointed out how important early diagnosis of VL is, as it has an impact on individual prognosis as well as on curtailing transmission. Individuals with a high probability of developing clinical disease might be treated sooner if given an intense follow-up scheme. Based on our data it is, therefore, tempting to promote the high-titre DAT, high–titer rK39 ELISA, and qPCR as markers to identify persons at high risk for clinical VL. However, the relatively low positive predictive value of the markers (ranging from 8.8% for qPCR over 10.4% for high DAT titres and 15.9% for rK39) warrants a word of caution. As there is no easy and absolutely safe treatment available, an attitude of watchful waiting is probably best at this time, observing those persons closely to detect the first clinical signs early enough. If one decided to treat all the qPCR positives or all the high titre DAT positives, about nine out of ten persons treated would be treated without reason, while for the rK39 this amounts to 8 out of ten. Combining tests does not add much to sensitivity. It should also be noted that incident VL cases that were missed at baseline, i.e. that did not show high-titre rK39 and DAT, did not arise until 6 months after the start of follow-up; half of them arose only after 18 months of follow-up or later. These subjects may well have been infected during the follow-up period. Importantly, recent reports from Bangladesh indicate that incorporating rKR95 and rTR18 with rK39 in serological tests conferred a sensitivity of 84% and may enable simple and accurate detection of asymptomatic infection in surveillance [32].

Finally, the potential epidemiological importance of the group of people with at least one positive marker of infection but no symptoms remains elusive. Are they all truly “infected”–i.e. latent carriers of *L*.*donovani*—with potential for transmission of the parasite or is this, more plausibly, a mixed group of i) very recently infected persons, ii) established latent carriers and iii). Immune persons who cleared their infection? It is hoped that the xenodiagnosis studies will shed some light on this question in the near future.

In conclusion, healthy persons living in VL endemic areas who have high antibody titers or test positive to qPCR have an increased probability to progress to VL disease. Such probability is not high enough to merit prophylactic treatment but carefull follow-up is warranted as most of those who do progress to disease eventually do so within the first 6 months.

## Supporting information

S1 Checklist STROBE checklist. (DOCX)

S1 Table Schematic of study design. (DOCX)

## References

[1] Alvar J, Velez ID, Bern C, Herrero M, Desjeux P, Cano J, et al Leishmaniasis worldwide and global estimates of its incidence. PLoS One. 2012;7(5):e35671 10.1371/journal.pone.0035671 22693548 PMC3365071

[2] National Road Map For Kala-Azar Elimination, Directorate of National Vector Borne Disease Control Program (NVBDCP), Directorate General of Health Services, Minister of Health & Family Welfare New Delhi 2014.

[3] Singh OP, Hasker E, Boelaert M, Sunadr S. Elimination of visceral leishmaniasis on the Indian Subcontinent Lancet Infect Dis. 2016;12;16(12):e304–e309. 10.1016/S1473-3099(16)30140-2 27692643 PMC5177523

[4] Singh OP, Hasker E, Sacks D, Boelaert M, Sundar S. Asymptomatic Leishmania Infection: A New Challenge for Leishmania Control. Clin Infect Dis. 2014 Epub ciu102 10.1093/cid/ciu102 . 24585564 PMC4001287

[5] Bern C, Haque R, Chowdhury R, Ali M, Kurkjian KM, Vaz L, et al The epidemiology of visceral leishmaniasis and asymptomatic leishmanial infection in a highly endemic Bangladeshi village. Am J Trop Med Hyg. 2007;76(5):909–14. . 17488915

[6] Ostyn B, Gidwani K, Khanal B, Picado A, Chappuis F, Singh SP, et al Incidence of symptomatic and asymptomatic Leishmania donovani infections in high-endemic foci in India and Nepal: a prospective study. PLoS Negl Trop Dis. 2011;5(10):e1284 10.1371/journal.pntd.0001284 21991397 PMC3186756

[7] Stauch A, Sarkar RR, Picado A, Ostyn B, Sundar S, Rijal S, et al Visceral leishmaniasis in the Indian subcontinent: modelling epidemiology and control. PLoS Negl Trop Dis. 2011;5(11):e1405 10.1371/journal.pntd.0001405 22140589 PMC3226461

[8] Le Rutte EA, Coffeng LE, Bontje DM, Hasker EC, Postigo JA, Argaw D, et al Feasibility of eliminating visceral leishmaniasis from the Indian subcontinent: explorations with a set of deterministic age-structured transmission models. Parasit Vectors. 2016;1 19;9:24 10.1186/s13071-016-1292-0 26787302 PMC4717541

[9] Chappuis F, Sundar S, Hailu A, Ghalib H, Rijal S, Peeling RW, et al Visceral leishmaniasis: what are the needs for diagnosis, treatment and control? Nat Rev Microbiol. 2007;5(11):873–82. 10.1038/nrmicro1748 . 17938629

[10] Hirve S, Boelaert M, Matlashewski G, Mondal D, Arana B, Kroeger A, et al Transmission Dynamics of Visceral Leishmaniasis in the Indian Subcontinent—A Systematic Literature Review. PLoS Negl Trop Dis. 2016;8 4;10(8):e0004896 10.1371/journal.pntd.0004896 27490264 PMC4973965

[11] Medley GF, Hollingsworth TD, Olliaro PL, Adams ER. Health-seeking behaviour, diagnostics and transmission dynamics in the control of visceral leishmaniasis in the Indian subcontinent. Nature. 2015;12 3;528(7580):S102–8. 10.1038/nature16042 26633763

[12] Hasker E, Kansal S, Malaviya P, Gidwani K, Picado A, Singh RP, et al Latent infection with Leishmania donovani in highly endemic villages in Bihar, India. PLoS Negl Trop Dis. 2013;7(2):e2053 10.1371/journal.pntd.0002053 23459501 PMC3573094

[13] Chapman LA, Dyson L, Courtenay O, Chowdhury R, Bern C, Medley GF, et al Quantification of the natural history of visceral leishmaniasis and consequences for control. Parasite and Vectors. 2015;10 22;8:521 10.1186/s13071-015-1136-3 26490668 PMC4618734

[14] Consortium LeishGEN, Fakiola M, Strange A, Cordell HJ, Miller EN, Pirinen M, et al Common variants in the HLA-DRB1-HLA-DQA1 HLA class II region are associated with susceptibility to visceral leishmaniasis. Nat Genet. 2013;45(2):208–13. 10.1038/ng.2518 23291585 PMC3664012

[15] Hasker E, Malaviya P, Gidwani K, Picado A, Ostyn B, Kansal S, et al Strong association between serological status and probability of progression to clinical visceral leishmaniasis in prospective cohort studies in India and Nepal. PLoS Negl Trop Dis. 2014;8(1):e2657 10.1371/journal.pntd.0002657 24466361 PMC3900391

[16] Singh OP, Singh B, Chakravarty J, Sundar S. Current challenges in treatment options for visceral leishmaniasis in India: a public health perspective. Infect Dis Poverty. 2016;3 8;5:19 10.1186/s40249-016-0112-2 26951132 PMC4782357

[17] el Harith A, Kolk AH, Leeuwenburg J, Muigai R, Huigen E, Jelsma T, et al Improvement of a direct agglutination test for field studies of visceral leishmaniasis. J Clin Microbiol. 1988;26(7):1321–5. 3410946 10.1128/jcm.26.7.1321-1325.1988 PMC266601

[18] Jacquet D, Boelaert M, Seaman J, Rijal S, Sundar S, Menten J, et al Comparative evaluation of freeze-dried and liquid antigens in the direct agglutination test for serodiagnosis of visceral leishmaniasis (ITMA-DAT/VL). Trop Med Int Health. 2006;11(12):1777–84. 10.1111/j.1365-3156.2006.01743.x . 17176341

[19] Khanal B, Rijal S, Ostyn B, Picado A, Gidwani K, Menten J, et al Serological markers for leishmania donovani infection in Nepal: Agreement between direct agglutination test and rK39 ELISA. Trop Med Int Health. 2011;15(11):1390–4. . 21998875 10.1111/j.1365-3156.2010.02631.x

[20] Gidwani K, Jones S, Kumar R, Boelaert M, Sundar S. Interferon-gamma release assay (modified QuantiFERON) as a potential marker of infection for Leishmania donovani, a proof of concept study. PLoS Negl Trop Dis. 2011;5(4):e1042 10.1371/journal.pntd.0001042 21526219 PMC3079582

[21] Singh OP, Gidwani K, Kumar R, Nylen S, Jones SL, Boelaert M, et al Reassessment of immune correlates in human visceral leishmaniasis as defined by cytokine release in whole blood. Clin Vaccine Immunol. 2012;19(6):961–6. 10.1128/CVI.00143-12 22539471 PMC3370446

[22] Sudarshan M, Singh T, Singh AK, Chourasia A, Singh B, Wilson ME, et al Quantitative PCR in epidemiology for early detection of visceral leishmaniasis cases in India. PLoS Negl Trop Dis. 2014;11;8(12):e3366 10.1371/journal.pntd.0003366 25503103 PMC4263468

[23] Weirather JL, Jeronimo SM, Gautam S, Sundar S, Kang M, A KM, et al Serial quantitative PCR assay for detection, species discrimination, and quantification of Leishmania spp. in human samples. J Clin Microbiol. 2011;49(11):3892–904. 10.1128/JCM.r00764-11 22042830 PMC3209110

[24] Ali A, Ashford RW. Visceral leishmaniasis in Ethiopia. II. Annual leishmanin transformation in a population. Is positive leishmanin reaction a life-long phenomenon? Ann Trop Med Parasitol. 1993;87(2):163–7. . 8561523 10.1080/00034983.1993.11812750

[25] Hailu A, Berhe N, Sisay Z, Abraham I, Medhin G. Seroepidemiological and leishmanin skin test surveys of visceral leishmaniasis in south and southwest Ethiopia. Ethiop Med J. 1996;34(1):11–23. . 8674496

[26] Schaefer KU, Kurtzhals JA, Kager PA, Gachihi GS, Gramiccia M, Kagai JM, et al Studies on the prevalence of leishmanin skin test positivity in the Baringo District, Rift Valley, Kenya. Am J Trop Med Hyg. 1994;50(1):78–84. . 8304576 10.4269/ajtmh.1994.50.78

[27] Das VN, Siddiqui NA, Verma RB, Topno RK, Singh D, Das S, et al Asymptomatic infection of visceral leishmaniasis in hyperendemic areas of Vaishali district, Bihar, India: a challenge to kala-azar elimination programmes. Trans R Soc Trop Med Hyg. 2011;105(11):661–6. 10.1016/j.trstmh.2011.08.005 . 21945327

[28] Costa CH, Stewart JM, Gomes RB, Garcez LM, Ramos PK, Bozza M, et al Asymptomatic human carriers of Leishmania chagasi. Am J Trop Med Hyg. 2002;66(4):334–7. . 12164285 10.4269/ajtmh.2002.66.334

[29] Bhattarai NR, Van der Auwera G, Khanal B, De Doncker S, Rijal S, Das ML, et al PCR and direct agglutination as Leishmania infection markers among healthy Nepalese subjects living in areas endemic for Kala-Azar. Trop Med Int Health. 2009;14(4):404–11. 10.1111/j.1365-3156.2009.02242.x . 19228350

[30] le Fichoux Y, Quaranta JF, Aufeuvre JP, Lelievre A, Marty P, Suffia I, et al Occurrence of Leishmania infantum parasitemia in asymptomatic blood donors living in an area of endemicity in southern France. J Clin Microbiol. 1999;37(6):1953–7. 10325353 10.1128/jcm.37.6.1953-1957.1999 PMC84994

[31] Sundar S, Singh OP. Molecular diagnosis of visceral leishmaniasis. *Mol*. *Diag Ther*. 2018; 22 (4):443–457. 10.1007/s40291-018-0343-y . 29922885 PMC6301112

[32] Vallur AC, Reinhart C, Mohamath R, Goto Y, Ghosh P, Mondal D, Duthie MS, Reed SG. Accurate Serodetection of Asymptomatic Leishmania donovani Infection by Use of Defined Antigens. J Clin Microbiol. 2016 4;54(4):1025–30. 10.1128/JCM.02620-15 . 26842701 PMC4809943

