# Supplementary material for: Determinants for progression from asymptomatic infection to symptomatic visceral leishmaniasis: A cohort study
Source: PLoS Negl Trop Dis. 2019 Mar 27;13(3):e0007216. doi: 10.1371/journal.pntd.0007216 (PMC6453476; doi:10.1371/journal.pntd.0007216)
Supplement: S1 Checklist — (DOCX) [file pntd.0007216.s001.docx]

STROBE Statement—Checklist of items that should be included in reports of ***cohort studies***

|  | Item No | Recommendation |
| --- | --- | --- |
| **Title and abstract** | 1 | (*a*) Indicate the study’s design with a commonly used term in the title or the abstract  - **Indicated in the title and abstract** |
|  |  | (*b*) Provide in the abstract an informative and balanced summary of what was done and what was found  **-Abstract contains a summary of methods, resulting odds ratios and discussion of significance (page 2)** |
| Introduction | | |
| Background/rationale | 2 | Explain the scientific background and rationale for the investigation being reported  **- It is found at pages 4 and 5** |
| Objectives | 3 | State specific objectives, including any prespecified hypotheses  **Included in Abstract (page 2) and Introduction (page 5)** |
| Methods | | |
| Study design | 4 | Present key elements of study design early in the paper  **Included in Abstract (page 2) and Materials and Methods section (pages 5-7)** |
| Setting | 5 | Describe the setting, locations, and relevant dates, including periods of recruitment, exposure, follow-up, and data collection  **Included in Methods section (pages 5)** |
| Participants | 6 | (*a*) Give the eligibility criteria, and the sources and methods of selection of participants. Describe methods of follow-up  **-Eligibility and selection procedures are described in both the abstract (page 2) and the Study population section (pages 6)** |
|  |  | (*b*) For matched studies, give matching criteria and number of exposed and unexposed  **-Matching criteria and number of exposed and unexposed subjected are included in the Study population sections (pages 6)** |
| Variables | 7 | Clearly define all outcomes, exposures, predictors, potential confounders, and effect modifiers. Give diagnostic criteria, if applicable  **-See Methods section (pages 5-7)** |
| Data sources/ measurement | 8* | For each variable of interest, give sources of data and details of methods of assessment (measurement). Describe comparability of assessment methods if there is more than one group  **-See Data Analysis section (pages 9-10)** |
| Bias | 9 | Describe any efforts to address potential sources of bias  **-It is found at pages 5-7.** |
| Study size | 10 | Explain how the study size was arrived at  **- is found at page 6, in the “Study population” section (pages 9-10)** |
| Quantitative variables | 11 | Explain how quantitative variables were handled in the analyses. If applicable, describe which groupings were chosen and why  **- It is found in the “Data Analysis” section (pages 9-10)** |
| Statistical methods | 12 | 1. Describe all statistical methods, including those used to control for confounding   **- It is found in the “Data Analysis” section (pages 9-10)** |
|  |  | (*b*) Describe any methods used to examine subgroups and interactions  **-Not applicable** |
|  |  | (*c*) Explain how missing data were addressed  **-Not applicable** |
|  |  | (*d*) If applicable, explain how loss to follow-up was addressed  **- It is found at page 10,11, in the “Result” section and Table-2** |
|  |  | (*e*) Describe any sensitivity analyses  **-Not applicable** |
| Results | | |
| Participants | 13* | (a) Report numbers of individuals at each stage of study—eg numbers potentially eligible, examined for eligibility, confirmed eligible, included in the study, completing follow-up, and analysed  **-See Results section (page 10) and Table-1 &2** |
|  |  | (b) Give reasons for non-participation at each stage  **-See Results section (page 10)** |
|  |  | (c) Consider use of a flow diagram  **-See Results section (page 10)** |
| Descriptive data | 14* | (a) Give characteristics of study participants (eg demographic, clinical, social) and information on exposures and potential confounders  **-The characteristics of study population (page-9-10) and in the Results section (page 10-11)** |
|  |  | (b) Indicate number of participants with missing data for each variable of interest  **-Not applicable** |
|  |  | (c) Summarise follow-up time (eg, average and total amount)  **-It is found inTable-2** |
| Outcome data | 15* | Report numbers of outcome events or summary measures over time  **-See Table 1&2 and Results section (page 10-12)** |
| Main results | 16 | (*a*) Give unadjusted estimates and, if applicable, confounder-adjusted estimates and their precision (eg, 95% confidence interval). Make clear which confounders were adjusted for and why they were included  **-It is found at pages 10 to 12 & in tables 1 and 2** |
|  |  | (*b*) Report category boundaries when continuous variables were categorized  **-It is found in Methods section (page 5-10)** |
|  |  | (*c*) If relevant, consider translating estimates of relative risk into absolute risk for a meaningful time period  **-Not applicable** |
| Other analyses | 17 | Report other analyses done—eg analyses of subgroups and interactions, and sensitivity analyses  **- Not applicable** |
| Discussion | | |
| Key results | 18 | Summarise key results with reference to study objectives  **-See Discussion section (page 12-14)** |
| Limitations | 19 | Discuss limitations of the study, taking into account sources of potential bias or imprecision. Discuss both direction and magnitude of any potential bias  **-See Discussion section (pages 12-14)** |
| Interpretation | 20 | Give a cautious overall interpretation of results considering objectives, limitations, multiplicity of analyses, results from similar studies, and other relevant evidence  **-See Discussion section (pages 12-14)** |
| Generalisability | 21 | Discuss the generalisability (external validity) of the study results  **-See Discussion section** |
| Other information | | |
| Funding | 22 | Give the source of funding and the role of the funders for the present study and, if applicable, for the original study on which the present article is based  **- It is included in the financial disclosure section of the online submission system.** |

*Give information separately for exposed and unexposed groups.

**Note:** An Explanation and Elaboration article discusses each checklist item and gives methodological background and published examples of transparent reporting. The STROBE checklist is best used in conjunction with this article (freely available on the Web sites of PLoS Medicine at http://www.plosmedicine.org/, Annals of Internal Medicine at http://www.annals.org/, and Epidemiology at http://www.epidem.com/). Information on the STROBE Initiative is available at http://www.strobe-statement.org.
