# Supplementary material for: Determinants for progression from asymptomatic infection to symptomatic visceral leishmaniasis: A cohort study
Source: PLoS Negl Trop Dis. 2019 Mar 27;13(3):e0007216. doi: 10.1371/journal.pntd.0007216 (PMC6453476; doi:10.1371/journal.pntd.0007216)
Supplement: S1 Table — (DOCX) [file pntd.0007216.s002.docx]

**Schematic of Study Design**:

**Year 1**

**Year 2**

- First round of house-to-house visits in 10,000 households (60,000 persons). Interview and clinical screening of all household members to identify at least 110 persons with VL over the past year. Ascertain case status by rK39 strip test.
- Take a systematic sample of all possible controls . Ascertain control status by rK39 strip test and interview
- Second round of house-to-house visits in 10,000 households (60,000 persons), same population as year 1. Interview and clinical screening of all household members to identify at least 110 persons with VL over the past year.
- Take a systematic sample of all possible controls (see above)

Continue to recruit subjects from new areas termed as Area-2 followed by collection of blood samples for marker analysis.

Following tests to be performed:

Quantiferon assay,

SNP/HLA genotyping

Buffy coat culture,q PCR,

HIV test.

.

**Year 2**

**Year 3**

**Year 4**

**Year 5**

First sero-survey (DAT, rK39 ELISA) of 12,000 residents of high-incidence villages to identify at least 6,000 susceptibles.

Second sero-survey (DAT, rK39 ELISA) of 6,000 susceptibles to identify true incident infections for enrolment in cohort study

+ m.

Follow up of incident infection subject

Follow up of incident infection subject

**PHAS**

**E**

**I**

**PHAS**

**E II**
